# Clinico-Microbiological Profile and Clinical Predictor of Urinary Tract Infection in Children: A Single-Center Study From Himalayan Foothills

**DOI:** 10.7759/cureus.33289

**Published:** 2023-01-03

**Authors:** Vinod Kumar, R. K. Naresh Singh, Prashant Kumar Verma, Nowneet Kumar Bhat, Yash Shrivastava, Enono Yhoshu, Mohit Bhatia, Swathi Chacham

**Affiliations:** 1 Department of Pediatrics, All India Institute of Medical Sciences, Rishikesh, Rishikesh, IND; 2 Department of Pediatric Surgery, All India Institute of Medical Sciences, Rishikesh, Rishikesh, IND; 3 Department of Microbiology, All India Institute of Medical Sciences, Rishikesh, Rishikesh, IND; 4 Department of Pediatrics, All India Institute of Medical Sciences, Rishikesh, IND

**Keywords:** uti, empirical antibiotic, children, antibiotic susceptibility, culture positive

## Abstract

Background: Urinary tract infection (UTI) in children is one of the commonest bacterial infections seen in the pediatric population. Clinical presentation ranges from fever with or without focus and isolation of microbiological agents streamline the treatment. Moreover, local/regional microbial profiles are helpful in antibiotic selection, we conducted a study to assess the prevalence of urine culture positivity in a suspected case of UTI. In addition, antibiotic susceptibility patterns and ultrasonography (USG) finding in culture-positive patients were also studied.

Methods and materials: It is a prospective observational study comprising symptomatic children aged one month to 18 years presenting to the outpatient department (OPD), inpatient department (IPD), and the emergency department of Pediatrics with UTI during the period of September 2019 to September 2020. The recorded variables were demographic, clinical presentation, anthropometry, physical examination, blood biochemistry, and outcome. Urine samples were collected and processed as per standard protocols. USG was done for all culture-positive children. Data were presented as frequency, mean (SD) and parametric and non-parametric data were analyzed by Wilcoxon-Mann-Whitney U Test, Chi-Squared Test, or Fisher's Exact Test.

Results: Of the total 354 children, 202 (57.1%) were male and the prevalence of UTI was 64 (18.1%). E. coli (70.3%) was the commonest isolated organism followed by Klebsiella spp (15.6%) and Pseudomonas spp (7%) respectively. The mean (SD) age (months) of presentation of symptoms was significantly lower in culture-positive children as compared to [ 83.49 (58.96) vs 110.10 (58.60); p=0.001] culture-negative children. Fever (96.6%) followed by dysuria (20.1%) were the most common symptoms presented for UTI however dysuria (p=0.003), pus cells (p<0.0001), and RBCs (p=0.002) were significantly present in culture positive children. This study shows increased resistance to third generation of cephalosporins. This study revealed significant differences among various groups (organism growth in positive culture) and the Antibiotic susceptibility test (AST) with a p-value of <0.001.

Conclusion: The prevalence of culture-positive UTI was similar to the reported literature and the presence of fever, dysuria, pus cells, and RBC in urine were commonly observed in the lower age group. Amikacin can be used in suspected UTIs with cephalosporin as empirical antibiotics in the Himalayan Foothills region.

## Introduction

“Urinary tract infections (UTIs) are among the most common bacterial infections found in children" [1-3]. The prevalence of it varies with age. During infancy, the prevalence of male children is high, with a male: female ratio of 2.8-5.4:1. After infancy (>1 year), there is a high female prevalence with a male-to-female proportion of 1:10 [4]. The clinical course of a UTI varies with the age of the child. In infants < 3 months old, symptoms include lethargies, vomiting, irritability, fever, poor feeding, failure to thrive, pain in the abdomen, hematuria, and jaundice. While in children >3 months old, symptoms include fever, dysuria, frequency, and urinary urgency with flank pain. Escherichia coli is the most common organism that causes UTIs (> 75% of UTIs). Other microorganisms that cause UTIs include Klebsiella, Proteus, Enterobacter, Citrobacter, Enterococcus, Staphylococcus saprophyticus, etc. Fungal UTIs (Candida albicans) is often associated with continuous antibiotic treatment, urethral catheterization, or immunosuppression.

UTI in children may manifest with a plethora of symptoms that may be vague, variable, and making its clinical diagnosis very challenging. Fever is the most common symptom. Sometimes, a child may present with sepsis or fever without specific symptoms. Prompt diagnosis and focused treatment reduce the risk of renal scarring and other complications. For this purpose, an empirical antibiotic is usually prescribed even before the culture results are accessible. Globally, antibiotic resistance is increasing [5]. Inappropriate use of antibiotics by people, factories, and farms, poor sanitation and public health, and ineffective prevention and treatment of infections in healthcare settings are considered necessary reasons for the emergence and spread of antibiotic-resistant microorganisms [6].

Treatment of these children requires urine culture and appropriate antimicrobial sensitivity testing [7]. Unfortunately, studies on pediatric UTIs, especially from India, are very limited. In addition, organisms are isolated, and susceptibility patterns to different antimicrobial agents may vary from place to place. Over time in the same clinical setting, it is imperative to review these factors to achieve the best clinical outcome. From this perspective, the present study aims to assess the prevalence of urine culture positivity in a suspected case of UTI. In addition, antibiotic susceptibility patterns and USG finding in culture-positive patients.

## Materials and methods

This study was conducted from September 2019 to September 2020 in the Department of Pediatrics, AIIMS Rishikesh, a tertiary health center located in the foothills of the Himalayas (Uttarakhand, India). Ethical clearance from the Institutional Ethics Committee, AIIMS Rishikesh, was taken (AIIMS/IEC/20/410). The inclusion criteria were symptomatic children aged one month to 18 years presenting to OPD, IPD, and the emergency department of Pediatrics with UTI. The calculated sample size was 354, after taking the prevalence of UTI as 35.4% from the previous study [9] and a confidence interval of 95%. The recorded variables were demographic, clinical presentation, anthropometry, physical examination, blood biochemistry and outcome. A thorough history was taken, like fever onset and associated signs and symptoms such as vomiting, difficulty passing urine and stool, and other associated symptoms. A History was also obtained, including previous antibiotic intake, recent UTI, any known congenital anomalies, and known foci of infection. Complete general and physical examinations with necessary investigations were done on all the children. Blood investigations, urine examination (routine and culture), and antimicrobial susceptibility, for which a midstream urine sample was taken, were also carried out in all the children. In infants, for urine sample collection, either suprapubic aspiration or transurethral bladder catheterization was done. USG was done for all culture-positive children.

Sample collection

The first mid-stream urine sample was collected with asepsis precautions, in infants, either suprapubic aspiration or transurethral bladder catheterization was used to collect a urine sample. The urine samples obtained were sent for routine examination (URE) and urine culture and sensitivity (UCS). The samples which could not be analyzed immediately were stored in a refrigerator for up to 12-24 hours at 4°C. Repeat urine culture was done in contaminated cases, e.g., mixed growth of two or more pathogens or overgrowth of the periurethral flora (lactobacilli in healthy girls; enterococci in infants).

Processing of urine sample

The 10 mL of urine sample was then centrifuged at a speed of 2,000 rpm for approximately 3-5 minutes. The supernatant was decanted, and the remaining sediment was resuspended. A drop of this sediment was then analyzed under a microscope for the presence of pus cells or/and red blood cells. In this study, to define significant pyuria, we took the cut-off of >5 pus cells/HPF in centrifuged urine samples.

Method of urine culture

The urine sample was inoculated with Cystine-lactose-electrolyte-deficient (CLED) agar plates using a loop calibrated to 0.01 mL. To obtain the accurate number of colonies, plates were incubated under aerobic conditions for 24 hours at a temperature of 35-37°C. When a midstream urine sample is cultured, a colony counts greater than 100,000/mL of organisms from one bacterial species is considered significant. Specimens with negligible growth, mixed growth of two or more pathogens, or non-pathogen growth are not considered culture positive.

Positive urine culture

A positive urine culture is defined as the growth of >100,000^ ^colonies of a single uropathogen per mL of sample in a clean mid-stream of urine. This sample was used for antibiotic sensitivity testing using Kirby's Bauer disc diffusion per Clinical Laboratory Standard Institute (CLSI) Guidelines 2019. All these findings were recorded.

## Results

During the period of our study (12 months), we enrolled 354 cases whose ages ranged from 2-216 months (mean:105.29; median:106). Most of the patients were aged between six and 12 years of age (148;41.8%), followed by 13-18 years (97;27.4%), one to five years (89;25.1%) and one month to one year (20;5.6%). 202 (57.1%) and 152 (42.9%) patients were males and females, respectively. Patients presented with symptoms like fever (342;96.6%), dysuria (71;20.1%), constipation (25;7.1%), hematuria (20;5.6%), poor feeding/weight loss (20;5.6%), and pain-abdomen (19;5.4%), in decreasing order of frequency. Thus, fever was the most common symptom (342;96.6%).

Only 60 (16.9%) participants revealed significant findings on URM, and 64 (18.1%) participants had positive UCS. 294 (83.1%) and 290 (81.9%) participants had no significant findings on URM and negative UCS, respectively. Gender-wise distribution of the 64 culture-positive patients was uneven. Even though more cases were seen in males, there was still no significant correlation. The distribution is shown in Table 1.

**Table 1:** Clinical profile of children with UTI with positive and negative urine culture ***Significant at p<0.05, 1: Wilcoxon-Mann-Whitney U Test, 2: Chi-Squared Test, 3: Fisher's Exact Test

Parameters	Urine C/S		P-value
	Positive (n = 64)	Negative (n = 290)	
Age (Months)***	83.49 ± 58.95	110.10 ± 58.64	0.001^1^
Age Group***			0.020^2^
<1 Year	7 (10.9%)	13 (4.5%)	
1-5 Years	22 (34.4%)	67 (23.1%)	
6-12 Years	24 (37.5%)	124 (42.8%)	
13-18 Years	11 (17.2%)	86 (29.7%)	
Gender			0.126^2^
Male	42 (65.6%)	160 (55.2%)	
Female	22 (34.4%)	130 (44.8%)	
Presentation: Fever (Present)***	57 (89.1%)	285 (98.3%)	0.002^3^
Presentation: Dysuria (Present)***	49 (76.6%)	163 (56.2%)	0.003^2^
Presentation: Pain Abdomen (Present)	14 (21.9%)	57 (19.7%)	0.688^2^
Presentation: Hematuria (Present)	7 (10.9%)	12 (4.1%)	0.058^3^
Presentation: Poor Feeding/ Weight Loss (Present)	6 (9.4%)	14 (4.8%)	0.225^3^
Presentation: Constipation (Present)	8 (12.5%)	17 (5.9%)	0.100^3^
Urine RM: Significant Finding (Present)***	28 (43.8%)	32 (11.0%)	<0.001^2^
Urine RM: Pus Cells (Present)***	26 (40.6%)	22 (7.6%)	<0.001^2^
Urine RM: RBCs (Present)***	10 (15.6%)	12 (4.1%)	0.002^3^

Among these 64 culture-positive cases, the most common symptom was fever (57;89.06%), followed by dysuria (49;76.6%), pain in the abdomen (16;25%), constipation (10;15.6%), poor feeding/weight loss (8;12.5%) and hematuria (6;9.3%). The most common organism isolated among the culture-positive patients in our study was E. coli (45;70.3%), followed by Klebsiella (10;15.6%), Pseudomonas (3;4.7%), non-Albicans Candida (2;3.1%), Acinetobacter (1;1.6%), Enterococcus faecalis (1;1.6%), Methicillin-sensitive Staphylococcus aureus/MSSA (1;1.6%), and Proteus mirabilis (1;1.6%). Association between UCS and clinical parameters is shown in Table 1. Patients with positive-urine cultures were younger than patients with negative cultures (p-value: 0.001). Most of the urine culture-positive patients belonged to the age groups of 1-5 (34.4%) and 6-12 years (37.5%), while most of the culture-negative patients belonged to the age groups of 6-12 (42.8%) and 13-18 years (29.7%). Symptoms like fever and dysuria were present significantly more in culture positive than in culture-negative patients, with a p-value of 0.002 and 0.003, respectively. The presence of significant findings, pus cells, and RBCs on URM was found to be significantly more in urine culture positive than negative patients (p-value, <0.001, <0.001, and 0.002, respectively). Results of the antimicrobial sensitivity testing (AST) among the urine culture-positive patients have been shown in Table 2.

**Table 2:** Anti-microbial sensitivity testing (AST) pattern among the urine culture positive cases

AST	Frequency	Percentage
Amikacin+Meropenem	22	35.5%
3rd Generation Cephalosporins	16	25.8%
Amikacin+Piperacillin/Tazobactam	7	11.3%
Meropenem	5	8.1%
Amikacin	3	4.8%
Amikacin+Doxycyclin+Piperacillin/Tazobactam	2	3.2%
Linezolid	2	3.2%
Amikacin+Doxycyclin	1	1.6%
Cefepime	1	1.6%
None	1	1.6%
Tetracycline	1	1.6%
Vancomycin	1	1.6%
Total	62	100.0%

Most of the growing organisms showed sensitivity to amikacin, followed by the 3rd generation of cephalosporin. In our study, the most common organism identified was E. coli (45;70.3%), among whom 39 (86.6%), 10 (22.2%), four (8.8%), one (2.2%), and one (2.2%) were sensitive to amikacin, third generation cephalosporins, meropenem, vancomycin, and cefepime, respectively. The distribution of organisms and their respective sensitive patterns to antibiotics are shown in Table 3.

**Table 3:** Distribution of isolated organisms in positive urine culture and their sensitive pattern *All three also sensitive to Gentamycin R- resistance, S- sensitive, N- none, s/t- sensitive to.

Organisms	No	3rd generation cephalosporin	Amikacin	Meropenem	Vancomycin	Linezolid
Escherichia coli	45	S ;10 (22.2%)	S; 39 (86.6%)	S;4(8.8%)	S; 1(2.2%)	N
Klebsiella	10	S; 3 (30%)	S; 9(90%)	S; 9(90%)	N	N
*Pseudomonas	3	S;3	S;3	S;3	N	N
Non-Albicans Candida	2	N	N	N	N	N
Acinetobacter	1	R	R	S	R	R
Enterococcus faecalis	1	R	R	R	S	R
MSSA	1	R	R	R	S	S
Proteus mirabilus	1	R	S	S	R	R

While analyzing the AST distribution pattern among isolated organisms in positive urine culture, we found a significant difference between the various groups regarding AST distribution (χ^2^ = 117.443, p = <0.001) Table 4.

**Table 4:** Association between organisms on urine C/S and AST (n = 62)

AST	Organism on Urine C/S								Chi-Squared Test	
	Escherichia coli	Klebsiella	Pseudomonas	Acinetobacter	Enterococcus faecalis	MSSA	Proteus mirabilis	Total	χ2	P-Value
Amikacin+Meropenem	18 (40.0%)	3 (30.0%)	0 (0.0%)	0 (0.0%)	0 (0.0%)	0 (0.0%)	1 (100.0%)	22 (35.5%)	117.443	<0.001
3rd Generation Cephalosporins	13 (28.9%)	3 (30.0%)	0 (0.0%)	0 (0.0%)	0 (0.0%)	0 (0.0%)	0 (0.0%)	16 (25.8%)		
Amikacin+Piperacillin/Tazobactam	4 (8.9%)	2 (20.0%)	1 (33.3%)	0 (0.0%)	0 (0.0%)	0 (0.0%)	0 (0.0%)	7 (11.3%)		
Meropenem	4 (8.9%)	0 (0.0%)	0 (0.0%)	1 (100.0%)	0 (0.0%)	0 (0.0%)	0 (0.0%)	5 (8.1%)		
Amikacin	2 (4.4%)	0 (0.0%)	1 (33.3%)	0 (0.0%)	0 (0.0%)	0 (0.0%)	0 (0.0%)	3 (4.8%)		
Amikacin+Doxycyclin+Piperacillin/Tazobactam	2 (4.4%)	0 (0.0%)	0 (0.0%)	0 (0.0%)	0 (0.0%)	0 (0.0%)	0 (0.0%)	2 (3.2%)		
Linezolid	0 (0.0%)	0 (0.0%)	0 (0.0%)	0 (0.0%)	1 (100.0%)	1 (100.0%)	0 (0.0%)	2 (3.2%)		
Amikacin+Doxycyclin	0 (0.0%)	1 (10.0%)	0 (0.0%)	0 (0.0%)	0 (0.0%)	0 (0.0%)	0 (0.0%)	1 (1.6%)		
Cefepime	1 (2.2%)	0 (0.0%)	0 (0.0%)	0 (0.0%)	0 (0.0%)	0 (0.0%)	0 (0.0%)	1 (1.6%)		
None	0 (0.0%)	0 (0.0%)	1 (33.3%)	0 (0.0%)	0 (0.0%)	0 (0.0%)	0 (0.0%)	1 (1.6%)		
Tetracycline	0 (0.0%)	1 (10.0%)	0 (0.0%)	0 (0.0%)	0 (0.0%)	0 (0.0%)	0 (0.0%)	1 (1.6%)		
Vancomycin	1 (2.2%)	0 (0.0%)	0 (0.0%)	0 (0.0%)	0 (0.0%)	0 (0.0%)	0 (0.0%)	1 (1.6%)		
Total	45 (100.0%)	10 (100.0%)	3 (100.0%)	1 (100.0%)	1 (100.0%)	1 (100.0%)	1 (100.0%)	62 (100.0%)		

In children with positive urine culture (n=68), a USG scan was done, which showed only eight children had significant findings, out of whom six patients (75%) were male, and two (25%) patients were female. One of the two females had a bladder mass, while the other one had PUJ obstruction with hydronephrosis. Overall, the presence of the posterior urethral valve (2;25%) was the most common finding, followed by the presence of bladder mass, bladder outlet obstruction, posterior urethral valve+vesico-ureteral reflux/VUR, pelvic-ureteric junction/PUJ obstruction, obstruction+hydronephrosis, and pyelonephritis in one-one case each (12.5%).

## Discussion

UTIs are one of the most common infections found in children. They are also a major cause of morbidity, leading to long-term consequences such as hypertension and renal failure. Hence, early diagnosis, identification of antibiotic sensitivity patterns, and adequate treatment of UTIs are important to avoid unnecessary financial burdens on patients and prevent the development of long-term complications.

Among 354 children with suspected UTI, 57.1% (202) were male, and 42.9% (152) were female, with a male-to-female ratio of 1.3:1 majority of the children were within the age group between six and 12 years. Among 354 patients, 64 (18.1%) patients showed positive urine cultures. Similarly, Badhan et al., Gupta et al. and Taneja et al. also reported a urine culture positivity of 26.7, 35.5, and 28.3%, respectively [8-10]. Our study also found fever as the most common presenting symptom, which accounts for 89% of all culture-positive patients, followed by dysuria (76.6%) and pain in the abdomen 25%, almost similar in males and females and similar to most of the previous studies done. Among culture-positive cases, 15.6%, 12.5%, and 9.3% presented with constipation, poor feeding, and hematuria, respectively. The major organism isolated was E. coli, followed by Klebsiella and Pseudomonas in our study, consistent with Gupta et al. and Masika et al. [9,11]. Our study demonstrated E. coli in 70.3%, Klebsiella in 15.6%, Pseudomonas in 4.7%, and Acinetobacter spp. In 1.6% of children. In contrast to our finding, the study by Taneja et al. demonstrated Pseudomonas in 10.9% and Acinetobacter spp. 6.6% of children as major pathogens in PICU settings [10]. “With rising rates of antibiotic resistance among the organisms causing UTI, antibiotic stewardship is critically needed to improve outpatient /outpatient antibiotic use, including in outpatient clinics (primary care and specialty clinics) and emergency departments” [12]. In our study, 76% (49/64) of culture-positive cases were sensitive to empirical antibiotics (third generation cephalosporins and amikacin), 20.3% of patients (13/64) needed higher antibiotics (piperacillin-tazobactam, meropenem, linezolid, and vancomycin). In our study, 86.6% (39/45), 22.2% (10/45), and 8.8% (4/45) culture-positive cases of E. coli sensitive to amikacin, third generation cephalosporins, and meropenem, respectively, were found. While 90% (9/10) and 30% (3/30) culture-positive cases for Klebsiella sensitive to amikacin and third generation cephalosporins, respectively. All three culture positives for Pseudomonas showed susceptibility to most of the antibiotics used. In addition, one Acinetobacter culture positive was isolated and sensitive to only meropenem; one Enterococcus faecalis was sensitive to only vancomycin. Our study is similar to other studies in which amikacin is highly sensitive to E. coli, and there is an increased resistance to ceftriaxone or third generation cephalosporins [9,13,14]. The results of this study again reemphasized the need for judicious use of antibiotics based on local culture and sensitivity patterns. On USG, eight out of 47 culture-positive cases showed significant findings, with a risk of 17% of urinary anomalies in our study. This finding was similar to a study done by Ahmadzadeh et al., which showed 20% associated anomalies [15]. Our study diagnosed cases of eight urinary anomalies for which prophylactic antibiotics were started, and surgery was done for those who required surgical interventions.

The limitation of our study is that we could not perform repeat USG (advised due to non-cooperative children and nonspecific findings at first scan) in 17 urine culture-positive children, and the prevalence of recurrent UTI and associated complications due to the COVID-19 pandemic and loss to follow-up.

## Conclusions

In our study patients, similar to many other studies, we observe that fever is the most common symptom presented for UTI. E. coli is the most common organism isolated in UTIs. Increasing resistance to third generation cephalosporin, which we used as an empirical antibiotic, was also observed, suggesting that the selection of empirical antibiotics should be based on awareness of the local prevalence and antibiotic sensitivity of bacteria rather than on general guidelines. This issue highlights the importance of performing antibiotic susceptibility testing before blinded antibiotic therapy. Our study also shows that an ultrasound performed after a culture-proven UTI provides clinically important information about possible urinary and renal abnormalities, which would help early intervention and prevent potential complications.
